# Miscellany

**Published:** 1904-04

**Authors:** 


### MISCELLANY.

---

CIVIL SERVICE EXAMINATIONS FOR STATE AND COUNTY SERVICE.

THE State Civil Service Commission announces general examinations to be held April 19, 1904, including the following positions: apothecary in state hospitals and institutions, assistant in entomology in the state museum, bridge designer, bridge draughtsman, building inspector, foremen of earth and rock borings, health officer for the town of Coventry, physicians in state hospitals and institutions of both regular and homeopathic schools, stenographer in state hospitals and institutions.

General examinations for stenographers will be held from May 25 to June 30. Applications for these examinations must be made on or before April 14. Full particulars of the examinations and blank applications may be obtained by addressing the Chief Examiner of the Commission at Albany.

